# Oral Region Homologies in Paleozoic Crinoids and Other Plesiomorphic Pentaradial Echinoderms

**DOI:** 10.1371/journal.pone.0077989

**Published:** 2013-11-11

**Authors:** Thomas W. Kammer, Colin D. Sumrall, Samuel Zamora, William I. Ausich, Bradley Deline

**Affiliations:** 1 Department of Geology and Geography, West Virginia University, Morgantown, West Virginia, United States of America; 2 Department of Earth and Planetary Sciences, University of Tennessee, Knoxville, Tennessee, United States of America; 3 Department of Paleobiology, National Museum of Natural History, Smithsonian Institution, Washington, District of Columbia, United States of America; 4 School of Earth Sciences, The Ohio State University, Columbus, Ohio, United States of America; 5 Department of Geosciences, University of West Georgia, Carrollton, Georgia, United States of America; University of Maryland, United States of America

## Abstract

The phylogenetic relationships between major groups of plesiomorphic pentaradial echinoderms, the Paleozoic crinoids, blastozoans, and edrioasteroids, are poorly understood because of a lack of widely recognized homologies. Here, we present newly recognized oral region homologies, based on the Universal Elemental Homology model for skeletal plates, in a wide range of fossil taxa. The oral region of echinoderms is mainly composed of the axial, or ambulacral, skeleton, which apparently evolved more slowly than the extraxial skeleton that forms the majority of the body. Recent phylogenetic hypotheses have focused on characters of the extraxial skeleton, which may have evolved too rapidly to preserve obvious homologies across all these groups. The axial skeleton conserved homologous suites of characters shared between various edrioasteroids and specific blastozoans, and between other blastozoans and crinoids. Although individual plates can be inferred as homologous, no directly overlapping suites of characters are shared between edrioasteroids and crinoids. Six different systems of mouth (peristome) plate organization (Peristomial Border Systems) are defined. These include four different systems based on the arrangement of the interradially-positioned oral plates and their peristomial cover plates, where PBS A1 occurs only in plesiomorphic edrioasteroids, PBS A2 occurs in plesiomorphic edrioasteroids and blastozoans, and PBS A3 and PBS A4 occur in blastozoans and crinoids. The other two systems have radially-positioned uniserial oral frame plates in construction of the mouth frame. PBS B1 has both orals and uniserial oral frame plates and occurs in edrioasterid and possibly edrioblastoid edrioasteroids, whereas PBS B2 has exclusively uniserial oral frame plates and is found in isorophid edrioasteroids and imbricate and gogiid blastozoans. These different types of mouth frame construction offer potential synapomorphies to aid in parsimony-based phylogenetics for exploring branching order among stem groups on the echinoderm tree of life.

## Introduction

Recent attempts to place early Paleozoic echinoderms into a comprehensive phylogenetic hypothesis have been hampered by the lack of a rigorous framework of underlying homologies with which to construct phylogenetic character matrices. Many advances have been made during the past few decades, notably the Axial-Extraxial Theory (EAT) [1], [2], that hypothesizes homology based on presumed embryonic origins of body regions and organ systems. Because EAT homologies are relatively coarse and difficult to reconcile with many Paleozoic taxa, this scheme has the greatest utility in phylogeny inference only for the most phylogenetically deep character states [3], [4]. The Universal Elemental Homology (UEH) model [3], [4] assigns hypotheses of homology at the level of individual plate elements, recognizing deep homologies of the ambulacral and peristomial systems in blastozoans and, therefore, is useful through several taxonomic levels. Here, we demonstrate how the blastozoan UEH model can be expanded into crinoids and edrioasteroids, suggesting how these homologies may be used to explore relationships among these three groups.

Universal Elemental Homology emphasizes the ambulacral or *axial* skeleton of Mooi and David [1], [2]. By combining ambulacral homology using the Carpenter system [5] with the homology of plate types in the peristomial border and ambulacral system, UEH identifies homology at a fine scale. This allows high precision in character description for subsequent analysis of echinoderm phylogeny. Such an approach is needed to infer phylogenetic relationships among extinct stem-group taxa within the echinoderm tree of life. These are the plesiomorphic pentaradial echinoderms: edrioasteroids, blastozoans, and crinoids (including extant Articulata). Excluded from this analysis are the extinct non-pentaradial echinoderms such as homalozoans and helicoplacoids, plus all eleutherozoans, which include all living echinoderms except the articulate crinoids.

By their very nature molecular data and other strictly neontological approaches can only be used to study relationships within the crown clade [6] – i.e. the clade descended from the last common ancestor of all living taxa [7]. Techniques that infer clades based on modern taxa lead to a nested hierarchy of crown clades and total clades [8]. Extinct taxa branch throughout this hierarchy, but because fossil organisms are not sampled using neontological approaches, relationships among extinct stem taxa can only be evaluated using morphological data [6]. If we are to fully understand the relationships between crinoids and their potential sister groups, it will be necessary to utilize morphological data that captures branching order among major clades.

Here, we focus on major echinoderm groups that exhibit well-preserved oral regions including blastozoans [9], [10]: eocrinoids, paracrinoids, diploporans, and glyptocystitoid rhombiferans; crinoids: hybocrinids, cladids, and camerates; and edrioasteroid-grade echinoderms. Groups lacking preserved oral regions, e.g. protocrinoids [11] are not included in this analysis, although they will be included in later analyses that consider more comprehensive character sets. Characters of the ambulacral/axial skeleton are *functionally integrated* in the oral region. They are good candidates to trace homologies among taxa because they must have evolved in concert, rather than individually, to maintain feeding function. This reduces the likelihood for unrecognized homoplasy. Phylogenetic relationships inferred by axial skeleton characters may provide key insights for identifying homologies within the extraxial skeletons of crinoids and blastozoans.

The wide range of existing morphological terminology within the groups under consideration has resulted in a poor correlation between names for morphology and homology. The same names may be used for non-homologous elements, whereas some homologous elements bear several different names [3], [4]. To avoid this problem, we will use a uniform lexicon of terminology for oral region characters of blastozoans, crinoids, and edrioasteroids following the recommendations of Sumrall and Waters [4].

The results herein demonstrate that the characters of the oral region in various groups of blastozoans have homologues in both edrioasteroids and crinoids. It is not our intention to document the full range of oral characters in these groups but rather to show clear examples of shared homologies suitable for parsimony-based phylogenetic analysis. Although phylogenetic implications may be suggested, a detailed phylogenetic analysis has yet to be done and will be presented in future papers, especially with reference to the origin of crinoids and their subsequent evolution.

## Previous Studies

Phylogenetic relationships among crinoids and other echinoderm clades is poorly constrained, although various hypotheses have been put forward. Ideas have included crinoid origins from several blastozoan groups such as eocrinoid-grade echinoderms [12]; “rhombiferans” [13], [14]; an unknown common ancestor closer to Eleutherozoa, used here as the clade including the last common ancestor of Asteroidea, Ophiuroidea, Echinoidea and Holothuroidea and all of its descendants [1]; an unknown common ancestor shared with stylophorans and asteroids [15]; or within edrioblastoid edrioasteroids [16].

Stalked echinoderms are known as pelmatozoans. They represent an evolutionary grade characterized by the possession, at least plesiomorphically, of a stalk or pelma for attachment, which may or may not be homologous among groups [9], [17]–[20]. Pelmatozoa includes crinoids, which bear distinct feeding structures termed arms, and blastozoans, which bear small feeding appendages termed brachioles, have various types of respiratory structures, and exhibit holoperipheral plate growth [9]. Blastozoa is a very diverse, potentially non-monophyletic assemblage comprising several major groups (“eocrinoids”, “rhombiferans”, “diploporans”, paracrinoids, coronoids, and blastoids) and a host of smaller groups, all of whose relationships are not well understood [10], [12], [15], [17], [21].

Stalks, in general, are considered to be an evolutionary grade [20]. Even some edrioasteroids with stalks, such as edrioblastoids [22], or those with greatly elongated peduncles, such as the rhenopyrgids [23], might be considered as pelmatozoans, although Bather's [24] definition included only crinoids, “cystoids”, and blastoids. Stalks are constructed in many ways. Plating can be irregular, bearing plates with imbricate or adjacent sutures as in various eocrinoids [9]. In many groups plating is organized into more regularly-arranged stacked columnals in a column, or stem, with a wide range of morphologies, including variation in the number of stem meres [25]–[27]. Stalks can even change plating type proximally to distally along their length. Thus, there are ample reasons to suspect that the details of stalk construction are analogous and not homologous across clades of pelmatozoans [10], [20], although stalks, columns, or stems may all be derived from homologous imperforate extraxial skeleton [2]. Clearly, more work is needed in this area, but that is beyond the scope of the present study.

If the possession of a stalk does not necessarily indicate common ancestry among pelmatozoans, is there evidence that blastozoans and crinoids are more closely related than each are to other echinoderm classes? Sprinkle [9], [10] argued that crinoids (as crinozoans) and blastozoans were distinct subphyla having diverged very early during the history of echinoderms, certainly by the middle Cambrian when it appeared these two clades were well established by the crinoid *Echmatocrinus* and eocrinoid blastozoans, such as *Gogia*. However, *Echmatocrinus* has subsequently been reclassified as an octocoral [28], [29]; but see Sprinkle and Collins [30] for an alternative interpretation. Thus, the oldest known crinoids are from the Early Ordovician, when several major clades first appeared, including the Protocrinoida, Aethocrinea, Camerata, Hybocrinida, Disparida, and Cladida [11], [13], [14], [16], [31]–[33]. Only the Middle Ordovician Flexibilia and the Post-Paleozoic Articulata are absent, both of which are phylogenetically nested within Cladida [34], [35]. The great diversity of Early Ordovician crinoids, distributed across several different paleocontinents, suggests a crinoid origin in the Cambrian, where true crinoids have yet to be described.

Guensburg and Sprinkle have argued that crinoids evolved independently from the other stalked echinoderms, the blastozoans, because the two groups lack shared derived characters. In particular, Guensburg and Sprinkle [16] hypothesized that crinoids evolved from late Cambrian edrioblastoid edrioasteroids, based on a few characters argued to be synapomorphic.

The Extraxial/Axial Theory (EAT) of Mooi and David [1], [2] emphasizes the separate developmental trajectories of the axial and extraxial skeletons. The axial skeleton in living echinoderms is subject to more ontogenetic organizing principles than the extraxial skeleton [1], offering the potential for slower evolutionary change and, thus, less homoplasy. The Universal Elemental Homology model [3], [4] provided a theoretical framework for understanding the origin and fate of individual plates associated with the mouth frame (peristomial border), ambulacral flooring plate system, and cover plate system for most blastozoans, and this framework extends seamlessly into crinoids and edrioasteroids, as documented herein. Recently, Smith and Zamora [36] applied the UEH model to a middle Cambrian spiral-plated echinoderm to demonstrate it had the earliest-known pentaradial body plan.

Universal Elemental Homology begins with assigning homology to the shared ambulacra and the five distal ambulacra following the Carpenter system [5], [37]. Plesiomorphically pentaradiate echinoderms arrange ambulacra into a 2-1-2 system in which three ambulacra, A, shared BC, and shared DE join to form the peristome (mouth). Distal bifurcation of the shared ambulacra form the distal B, C, D, and E ambulacra [9], [38], [39]. This ambulacral symmetry is formed ontogenetically and is consistent, although often modified, throughout pentaradiate taxa [3], [39]–[41]. These homologies are further identified by the positioning of the hydropore and gonopore that is typically positioned in the proximal CD interambulacrum and the periproct (anus) that is typically positioned in the distal CD interray [39].

## Materials and Methods

All taxa studied and their geologic ages are listed in Table 1. These have all previously been described in the literature, with references cited in the figure captions and Table 1. All studied specimens were from research collections in the following museums or institutions (ordered by numbers of taxa included herein): U.S. National Museum of Natural History (USNM); Cincinnati Museum Center (CMCIP); Sam Noble Oklahoma Museum of Natural History, University of Oklahoma (OU); Department of Geoscience, University of Iowa (SUI); Paleontology Museum of Guizhou University, China (GM and GTBM); Museum of Comparative Zoology, Harvard University (MCZ); Natural History Museum, University of Oslo (PMO); Faculté des Sciences et Techniques Guéliz, Université Cadi Ayyad. Marrakech, Morocco (FSTG); Royal Ontario Museum (ROM); Geological Survey of Canada, Ottawa (GSC); and Buffalo Museum of Science, New York (BMS). Catalog numbers for all illustrated specimens are given in the figure captions. The original geographic locations of specimens studied is not pertinent to this study, but such information is readily available from the literature and the above museums or institutions.

**Table 1:** List of key taxa discussed or illustrated; order follows Table 3.

Genus	Group	Age	Figure or Reference
**EDRIOASTEROIDEA**			
*Kailidiscus*	Order unnamed	Middle Cambrian	Figures 4A, B, E, F, 5A
*Cambraster*	Stromatocystitida	Middle Cambrian	[47]
*Edriophus*	Edrioasterida	Middle Ordovician	Figures 4G, H, I, J, 5E
*Astrocystites*	Edrioblastida	Middle Ordovician	Figure 6
*Anedriophus*	Isorophida	Lower Ordovician	Figure 4K, L
*Isorophusella*	Isorophida	Upper Ordovician	[38] pls. 31–36
*Hypsiclavus*	Isorophida	Mississippian	Figure 5F
**BLASTOZOA**			
*Ascocystites*	Eocrinoidea, Ascocystitida	Middle Ordovician	[9] text-fig. 30; [70]
*Lepadocystis*	Rhombifera, Glyptocystitida	Upper Ordovician	Figure 1
*Quadrocystis*	Rhombifera, Glyptocystitida	Middle Ordovician	Figure 2C, F
*Protocrinites*	Diploporida, Glyptosphaeritida	Lower Ordovician	Figure 5B
*Rhopalocystis*	Eocrinoidea, Rhopalocystida	Lower Ordovician	Figure 2A, B, D, E
*Eumorphocystis*	Diploporida, Eumorphocystida	Middle Ordovician	Figure 2G, H
*Columbocystis*	Paracrinoidea	Middle Ordovician	Figure 2J, K
*Stephanocrinus*	Blastoidea, Coronoidea	Middle Silurian	Figures 2I, L, 5D
*Lepidocystis*	Eocrinoidea, Imbricata	upper Lower Cambrian	Figure 4M, N
*Sinoeocrinus*	Eocrinoidea, Gogiida	Middle Cambrian	Figure 4C, D
**CRINOIDEA**			
*Hybocrinus*	Hybocrinida	Middle Ordovician	Figure 3H, I
*Carabocrinus*	Cladida	Middle Ordovician	Figure 3A, G
*Palaeocrinus*	Cladida	Middle Ordovician	Figure 5C
*Illemocrinus*	Cladida	Upper Ordovician	Figure 3K, L
*Nuxocrinus*	Cladida	Middle Devonian	Figure 3B, C
*Onychocrinus*	Flexibilia	Mississippian	[54] pl. 67, figs. 1–10
*Cyttarocrinus*	Camerata	Middle Devonian	Figure 3Q, R
*Marsupiocrinus*	Camerata	Middle Silurian	Figure 3O, P
*Collicrinus*	Camerata	Mississippian	Figure 3D, J
*Elegantocrinus*	Camerata	Mississippian	Figure 3E, F
*Neoplatycrinus*	Camerata	Permian	Figure 3M, N

No permits were required for the described study, which complied with all relevant regulations. Permission to study specimens was obtained from all the above listed museums or institutions. Specimens were studied as follows: 1) direct observation and photography on site by one or more of the authors: USNM S1296, S1310, S1337, 305473; CMCIP 40478, 40480, 57349; OU 8972, 9127, 9179; SUI 97598, 134856; MCZ 628; ROM 45102; GSC 752; BMS E21032; 2) loan of original material: SUI 134869, 134871, 134872 to CDS; PMO A29122, A29124 to TWK; and 3) latex rubber copies freely provided for our use: GM 2103, GTBM 95265, FSTG/AA-BCBb-OI-25. Specimen SUI 134870 was donated by CDS to the University of Iowa.

Data were assembled by searching for well-preserved oral regions of stem-group echinoderms in the literature and museum collections, focusing on early Paleozoic edrioasteroids, blastozoans, and crinoids. If the mouth was at the surface with moveable cover plates, the oral region is an oral surface. If the mouth was covered by a field of fixed plates, the oral region is a tegmen and the mouth is subtegmenal. As used here, the oral region is the area including the plates of the peristomial border (mouth frame), recumbent ambulacra, and any associated interambulacral plates; or it is the same area covered by fixed plates of a tegmen. Its outer edge is bordered by a rigid thecal or calyx wall formed by either a peripheral rim, as in isorophid edrioasteroids; imbricate plating, as in *Kailidiscus*, or by rigid calyx plates as in blastozoans and crinoids. In general, well-preserved oral surfaces are very rare in crinoids (we estimate much less than 1 percent of specimens in museum collections), but oral surfaces are much more common in blastozoans. Oral surfaces are visible where 1) the oral surface is a major feature of the theca, as in disk-shaped edrioasteroids; 2) the theca, including the oral surface, was buried before disarticulation and was not crushed by lateral compression; and 3) the oral surface has not been covered by closed arms as is common in crinoids. Because of the elongate shape (especially if the column is attached) stalked echinoderm theca almost always fell on their side at death. Consequently, they were laterally compressed after burial, typically crushing the oral surface at the adoral end of the theca because it is perpendicular to the long axis of the theca. Because of their rigidity, crinoid tegmens are much more common and are even abundant in many camerate genera.

The 25 taxa treated in this paper are listed in Table 1, not including additional taxa mentioned in the text for comparative purposes. These taxa include six edrioasteroid-grade echinoderms, nine blastozoans, and nine crinoids. Although this is not a complete list of taxa with well-preserved oral regions, it includes taxa with a representative range of character states for recognizing homologies across groups.

## Results: Oral Region Morphology

### Standardized Terminology

#### Theca

The oral region is part of the theca. The body of a pelmatozoan, minus any appendages including arms, brachioles, and the column, is the theca. The terms theca and calyx have been used interchangeably in crinoids and blastozoans [9], [42], but for consistency we define them following Ubaghs [25]. In a blastozoan, the theca is divisible into the summit, or oral surface, and the calyx. The calyx is that part of the body between the oral surface, above, and the column, below, if present. In a crinoid, the theca is defined the same, but the calyx encompasses all the plates between where the arms become free, above, and the column, below. If the arms are free above the radial plates, the calyx is also an aboral cup. In edrioasteroid-grade echinoderms the theca is the body of the animal, which may be spatially-dominated by the oral surface; the term calyx is not used.

#### Summit

**Figure 1:**
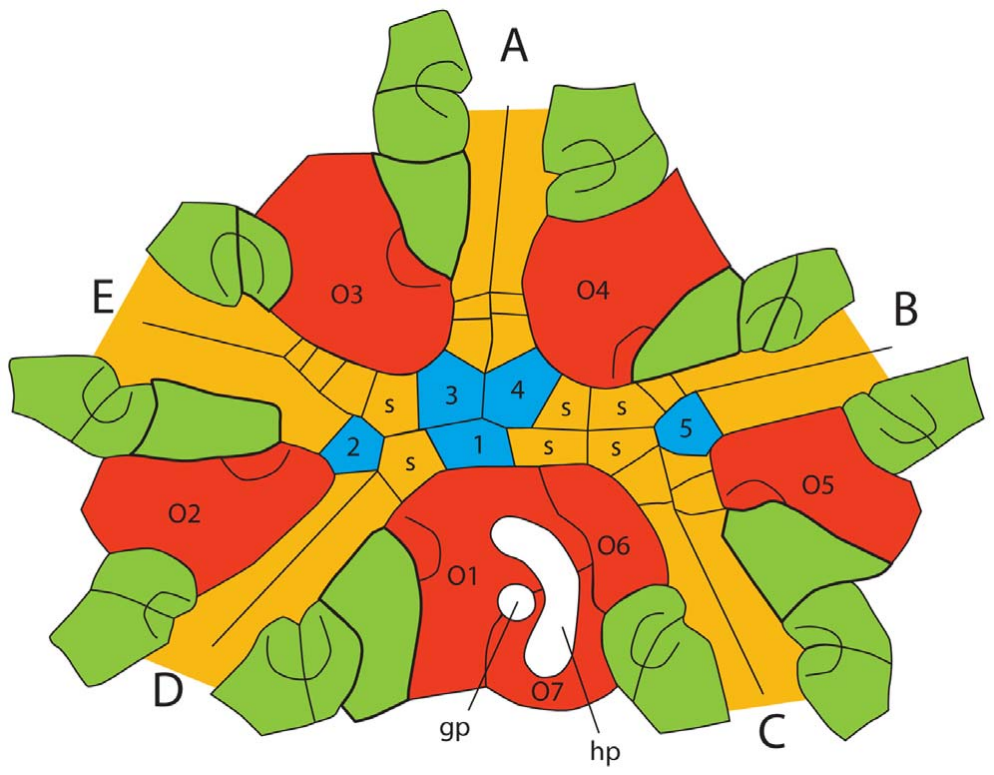
Universal elemental homology. Camera lucida drawing of the oral surface of the glyptocystitoid rhombiferan *Lepadocystis moorei* (Meek, 1871) [72] (CMCIP 57349) with plates colored according to Universal Elemental Homology. Red plates (O1–O7) are oral plates that form the border of the peristome. Blue plates (1–5) are primary peristomial cover plates (PPCP). Green plates are ambulacral floor plates (AFP), tan plates are ambulacral cover plates (ACP). A–E are the ambulacral designations based on the Carpenter [5] system. The mouth or peristome is located beneath PPCP1, 3, and 4. The gonopore and hydropore are gp and hp, respectively. S are shared cover plates of the shared ambulacra. (After [57]).

The summit is the mouth and associated structures at the top of the theca. Plesiomorphically in blastozoans, the summit has a centrally located peristome from which the ambulacra branch. The peristome can be bordered by a variety of plate types (see below) and plesiomorphically is covered by a moveable series of cover plates. Radiating out from the summit are typically five ambulacra arranged into a 2-1-2 configuration that are formed from trough-shaped floor plates covered by moveable cover plates, as in *Lepadocystis* (Figure 1).

Three different summit types, traditionally termed the tegmen, cap the calyx in crinoids. The plesiomorphic condition, present in hybocrinids, plesiomorphic cladids (as well as flexibles), and camerates, is an oral surface at the summit. An oral surface has an exposed mouth, is framed by the oral plates, and has moveable cover plates over the peristome and ambulacra. Herein, the term tegmen is restricted to a surface that covers a subtegmenal mouth. Possession of a tegmen occurs in more derived taxa. In typical camerates (and some cladids and disparids), the tegmen may be composed of fixed peristomial and ambulacral cover plates that are homologues of the moveable cover plates of blastozoans and plesiomorphic crinoids, or the plates of the tegmen may be completely undifferentiated with no apparent homologues. The latter situation is commonly associated with a large, rigid anal tube. Some cladids have convex tegmens composed of large, flexible anal sacs that completely cover the oral surface, whereas others may have rigid surfaces analogous with those of camerates.

#### Oral surface plates

**Figure 2:**
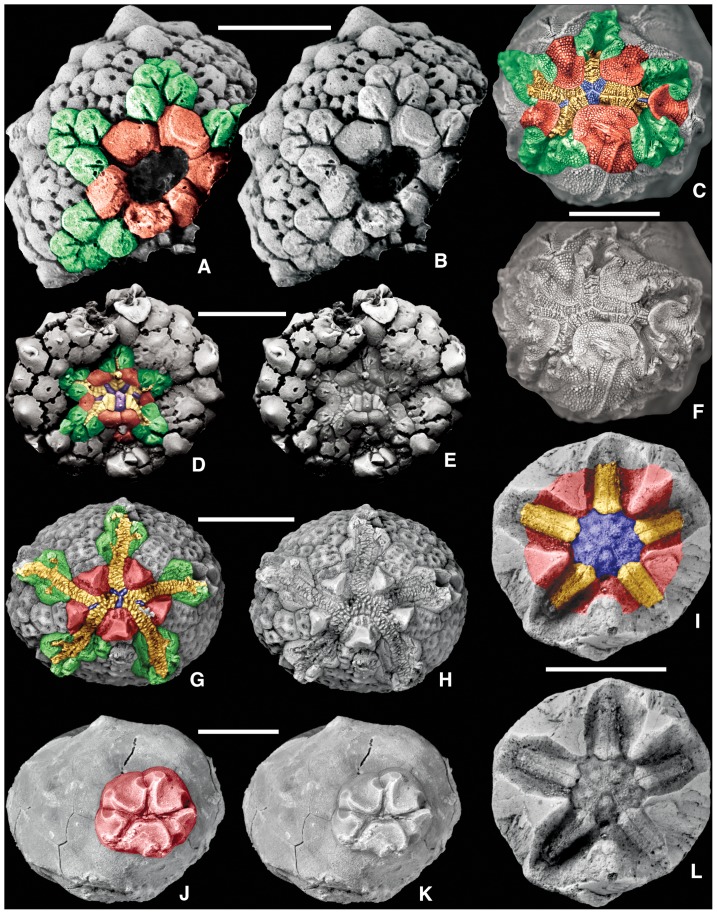
Homologous oral-region plates in more-derived blastozoans. Colored and uncolored views for plate comparison; color key as in Figure 1; geologic ages in Table 1. A,B, D, E. the eocrinoid *Rhopalocystis destombesi* Ubaghs, 1963 [73], A, B, latex cast of paratype PMO A29122 with only orals and floor plates preserved, D, E, latex cast of paratype PMO A29124 with all oral surface plates preserved; C, F. the glyptocystitoid *Quadrocystis graffhami* Sprinkle, 1982 [74], holotype OU 8972; G, H. the diploporan *Eumorphocystis multiporata* Branson and Peck, 1940 [75], SUI 97598; I, L. the coronoid *Stephanocrinus gemmiformis* Conrad, 1842 [76], SUI 134869; J, K. the paracrinoid *Columbocystis typica* Bassler, 1950 [77], SUI 134870. Scale bars: 5 mm (A–L).

Two blastozoan genera clearly display many of the oral surface plates and will serve as taxa for defining those characters and for comparison with other blastozoans, crinoids, and edrioasteroids. These model taxa are the Upper Ordovician glyptocystitoid *Lepadocystis moorei* (Figure 1), used by Sumrall and Waters [4], and the Lower Ordovician eocrinoid *Rhopalocystis destombesi* (Figure 2A, B, D, E). As much as possible, existing terminology for blastozoans [43], crinoids [25], and edrioasteroids [38] is conserved where confusion can be avoided. Characters demonstrated to be homologues are given the same names, if possible, following the lead of Sumrall [3] and Sumrall and Waters [4].

**Figure 3:**
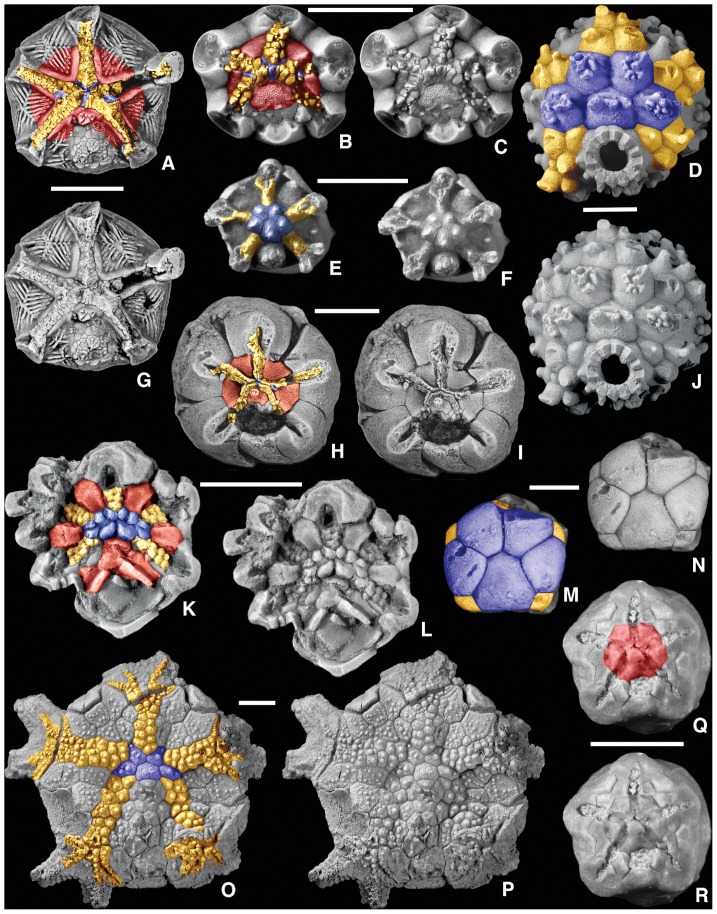
Homologous oral-region plates in crinoids. Colored and uncolored views for plate comparison; color key as in Figure 1; geologic ages in Table 1. A, G. the plesiomorphic cladid *Carabocrinus treadwelli* Sinclair, 1945 [78], OU 9127, note the lack of floor plates where the ambulacral cover plates are missing on the B-ray, and the hydropore in Oral 1; B, C. the plesiomorphic cladid *Nuxocrinus crassus* Whiteaves, 1887 [79], plesiotype, USNM 305473, note the madreporite in Oral 1; D, J. the camerate *Collicrinus yandelli* Owen and Shumard, 1850 [80], plesiotype, USNM S1337, note the absence of orals and the fixed PPCP and ACP that form the tegmen, along with interambulacral plates; E, F. a juvenile of the camerate *Elegantocrinus symmetricus* Wachsmuth and Springer, 1897 [81], plesiotype, USNM S1310, note the absence of orals and the fixed PPCP and ACP that form the tegmen, along with interambulacral plates; H, I. the plesiomorphic hybocrinid *Hybocrinus nitidus* Sinclair, 1945 [78], OU 9179, note the tiny PPCP plates homologous with those in Figure 2C, D,G and Figure 3A,B, also note the coelomic canals through the radial plates at the arm bases; K, L. the cladid *Illemocrinus amphiatus* Eckert, 1987 [82], paratype, ROM 45102, note that there are two PPCP at each interradial position, also note the coelomic canals through the radial plates at the arm bases; M, N. the neotenic camerate *Neoplatycrinus dilatatus* Wanner, 1916 [83], SUI 134856, note that the tegmen is composed exclusively of the five PPCP plus a single ACP in each ray; O, P. the camerate *Marsupiocrinus stellatus* (Troost *in* Wood, 1909 [84]), plesiotype, USNM S1296, note the absence of orals and the fixed PPCP and ACP that form the tegmen, along with interambulacral plates, distal ambulacra with moveable cover plates; Q, R. the camerate *Cyttarocrinus jewetti* Goldring, 1923 [85], BMS E21032, which is plesiomorphic in having visible orals, note that the PPCP and ACP are not preserved having fallen away as based on other specimens, but the orals have small prong-like extensions over the peristome. Scale bars: 5 mm (A–R).

**Figure 4:**
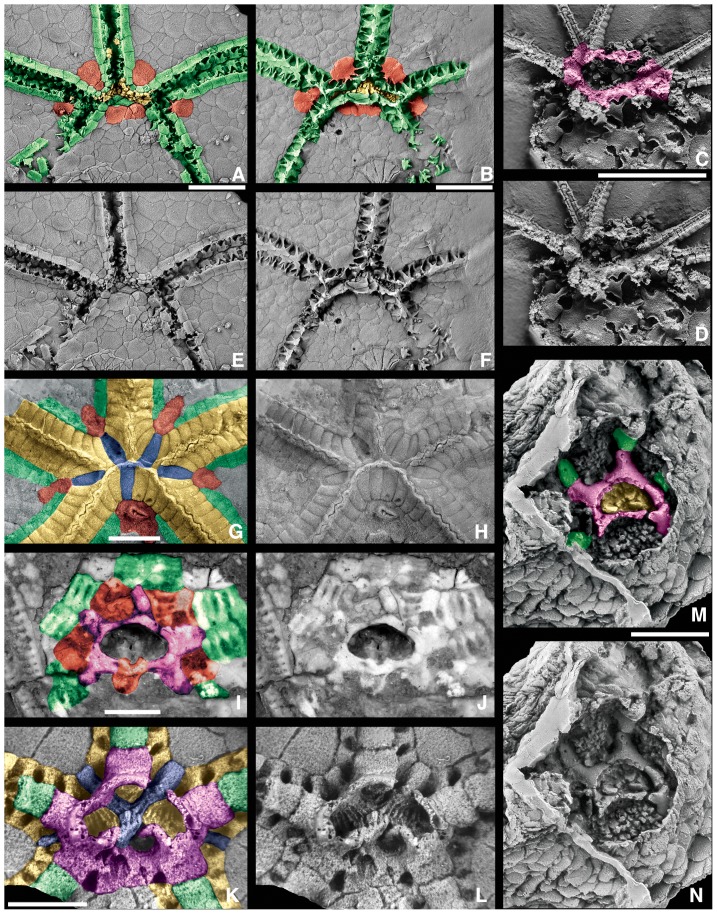
Homologous oral-region plates in edrioasteroids and less-derived blastozoans. Colored and uncolored views for plate comparison; color key as in Figure 1, except that purple plates are oral frame plates; geologic ages in Table 1. A, E,B, F. the plesiomorphic edrioasteroid *Kailidiscus chinensis* Zhao et al., 2010 [48], latex casts of paratype GM 2103, note the integrated interradial (IIP) plates, in red, judged to be oral plate homologues, A, E, exterior view, B,F, interior view; C, D. the gogiid blastozoan *Sinoeocrinus lui* Zhao et al., 1994 [86], latex cast of GTBM 95265, oral view with the radially positioned oral frame plates that give rise to brachioles; G, H, I, J. the edrioasterid edrioasteroid *Edriophus levis* Bather, 1914 [87], G, H, exterior view, note the oral plates, in red, CMCIP 40480, I, J, interior view, CMCIP 40478, note the orals in red and oral frame plates in purple form the peristomial border; K, L. the isorophid edrioasteroid *Anedriophus moroccoensis* Sumrall and Zamora, 2011 [53], FSTG/AA-BCBb-OI-25, interior view showing the radially positioned oral frame plates; M, N. the imbricate blastozoan *Lepidocystis* cf. *L. wanneri* Foerste, 1938 [88], latex cast of MCZ 628, interior view with the radially positioned oral frame plates that lead to floor plates. Scale bars: 5 mm (A, B, C, D, E–K, M, N), 2.5 mm (K, L).

Many oral surface characters in blastozoans (Figures 1 and 2) can be recognized in both crinoids (Figure 3) and edrioasteroids (Figure 4). These include orals (O), shared cover plates (SCP), primary peristomial cover plates (PPCP), ambulacral cover plates (ACP), hydropore through Oral 1, and position of the periproct in the CD interray. Previously in crinoids, orals were defined as either those plates forming the mouth circlet or peristomial border, or those plates fixed over the mouth [25]. Only those plates that form the peristomial border are considered true orals, homologous with blastozoan orals. Plates covering the mouth are now recognized as SCP and PPCP, whether moveable as in hybocrinids and some cladids, or fixed as in camerates. In camerates, the five fixed cover plates in the center of the tegmen are the PPCP. Because both orals and PPCP are present in individual crinoid taxa, these plate types are not homologous by the test of conjunction [44]. Previously termed orals, the PPCP circlet is one of the earliest circlets formed during crinoid ontogeny [45], [46].

In taxa where they occur, oral plates form the peristomial border. Orals are generally large plates positioned interradially and bear the ambulacral food groove along their depressed shared sutures. These plates are designated O1 through O7, with O1 positioned in the CD interambulacrum. Numbering proceeds clockwise around the oral surface to O5 in the BC interambulacrum. If present, O6 lies along the peristomial border in the CD interambulacrum and O7 lies slightly distal to O1 and O6 and is not in contact with the peristomial border (Figure 1). Commonly, O2 and O5 are smaller than O3 and O4 as they are added later in ontogeny in some taxa [4], [39], [41]. Furthermore, in many taxa, O2 and O5 are not in physical contact with the peristomial opening, whereas taxa with pseudo five-fold symmetry (sensu Sumrall and Wray [39]) have these plates sharing the peristomial border, such as in blastoids [4].

### Axial Skeleton in the Oral Region

#### Peristomial Border

The mouth (or peristome) is formed by the peristomial border. There are many different arrangements of plates around the peristome of non-eleutherozoan echinoderms that are presumably of great phylogenetic significance. The peristomial border may be composed of either oral plates (interradial elements) or oral frame plates (radial elements presumably derived from modified floor plates). In derived blastozoans and plesiomorphic crinoids, the peristomial border, or mouth frame, is composed of orals that are tightly sutured together. The food grooves run on top of the shared sutures between adjacent oral plates, and the cover plates articulate directly with the orals. In edrioasteroids the peristomial border may be constructed by both oral plates (integrated interradial plates, IIP) and biserial floor plates, as in *Kailidiscus* (Figure 4A, B, E, F); modified uniserial floor plates called oral frame plates, as in *Isorophusella* or *Anedriophus* (Figure 4K, L), or it may be loosely constructed by interradial mouth plates, as in *Cambraster* [47]. Based on position and size of the integrated interradial plates in *Kailidiscus* [48] and their equivalent interradial mouth plates in *Cambraster* [47], these plates are judged to be homologues of orals and will be subsequently referred to as orals.

**Figure 5:**
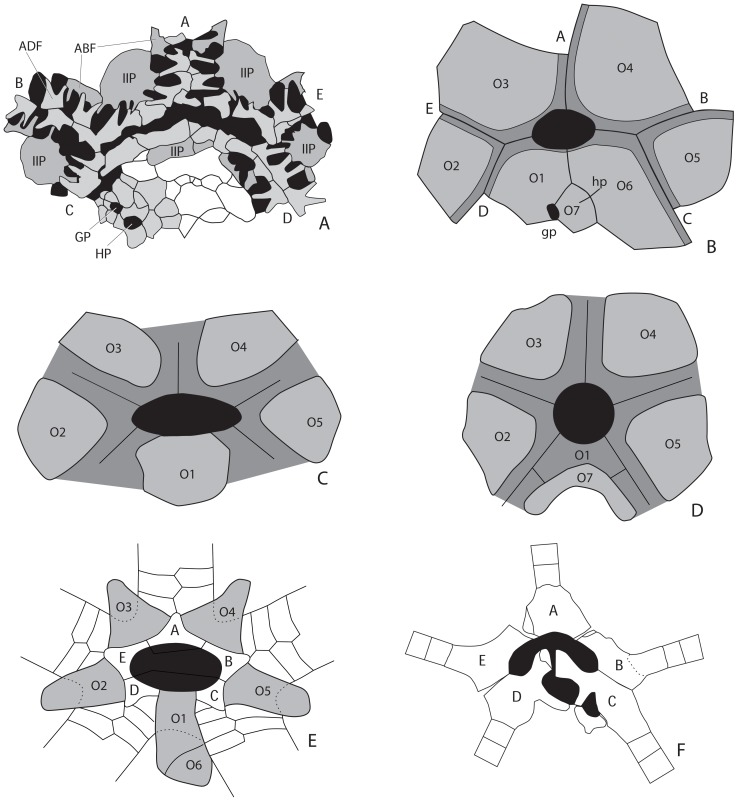
Examples of peristomial border systems (PBS) with cover plates removed to expose the mouth frame. A. PBS A1, interior view of *Kailidiscus* showing the mouth frame constructed by integrated interradial plates (IIP), or orals, and unfused biserial floor plates, black areas are the elongated peristome in the center and podial pores between plates, modified from [48]; B. PBS A2, the diploporan *Protocrinites* [4], note that orals 2 and 5 do not contact the peristome (black); C. PBS A3, the crinoid *Palaeocrinus*, note the oval peristome that had shared peristomial cover plates, modified from [89]; D. PBS A4, the coronoid *Stephanocrinus*, note the round peristome that was covered by only five primary peristomial cover plates, modified from [90]; E. PBS B1, the edrioasterid *Edriophus* with both oral plates (O1–O6) and oral frame plates (A–E), an interpretation based on Figures 4G, I; F. PBS B2, interior view of the isorophid *Hypsiclavus* showing oral frame plates (A–E) in line with uniserial floor plates, modified from [51].

Two fundamentally distinct Peristomial Border Systems (PBS) exist where the peristomial border is formed from interradially positioned oral plates (type A) or formed from radially positioned oral frame plates (type B) (Table 2, Figure 5). Further, four arrangements occur in PBS type A, and two arrangements occur in Type B. In PBS A1, the peristomial border is formed by both oral plates and un-fused, biserial inner ambulacral floor plates (Figure 5A). The peristome is covered by indistinct multi-tiered cover plates. This condition is recognized only in the Middle Cambrian edrioasteroids *Kailidiscus* and *Walcottidiscus* [48]. In *Kailidiscus*, the oral plates are not in contact with the peristomial opening and have podial pores suggesting their origin as fused outer floor plates [3], [48]. Thus, the orals of blastozoans and crinoids may have originated from fused outer floor plates.

**Table 2:** Characters of Peristomial Border Systems (PBS).

Characters	PBS A1	PBS A2	PBS A3	PBS A4	PBS B1	PBS B2
Border formed from combined IIP* and biserial floor plates; IIP have podial pores	X					
Border with IIP or oral plates only; O2 and O5 not in contact with peristome		X				
Border with oral plates only; all orals in contact with peristome			X	X		
Border formed from combined IIP* and uniserial oral frame plates					X	
Border formed exclusively from uniserial oral frame plates						X
Shared cover plates between PPCP** in BC and DE ambulacra	X	X	X		X	X
All 5 PPCP** meet in the center, no shared cover plates				X		

* IIP, integrated interradial plates ( = orals) in edrioasteroids.

** PPCP, primary peristomial cover plates.

In PBS A2, the peristome is bordered by oral plates and covered by small, distinct movable cover plates. This type is further characterized by having the smaller orals 2 and 5 outside of the peristomial border, as in the eocrinoid *Ascocystites* (Table 1), the glyptocystitoids *Lepadocystis* (Figure 1) and *Quadrocystis* (Figure 2C, F), and the diploporan *Protocrinites* (Figure 5B). As a result, the shared ambulacra each have a shared food groove. The cover plate system also has shared ambulacra characterized by a set of cover plates spanning the three central Primary Peristomial Cover Plates, PPCP 1, 3, and 4, and the lateral PPCP 2 and 5. Typically the CD oral is compound associated with the hydropore and or gonopore complex [4], [39].

In PBS A3, the peristome is bordered by oral plates and covered by small movable cover plates. Here all of the orals form the peristomial border as in the eocrinoid *Rhopalocystis* (Figure 2A, B, D, E), the diploporan *Eumorphocystis* (Figure 2G, H), or the hybocrinid crinoid *Hybocrinus* (Figure 3H, I). Each of the five food grooves enters the peristome separately (Figure 5C), but the shared ambulacra are expressed only in the cover plate system (Figure 2D, G). The set of shared cover plates spans the three central PPCP (1, 3, and 4) and the lateral PPCP (2 and 5), but without underlying shared food grooves. The CD oral may be singular or compound.

In PBS A4, all the oral plates define the peristomial border and each of the five food grooves enters the peristome independently (Figure 5D). However, the five PPCP meet in the center over the peristome with no shared cover plates to define the BC and DE ambulacra. Instead, the sutures of the PPCP articulate in 2-1-2- symmetry leaving a phylogenetic footprint of the plesiomorphic condition [39]. PBS A4 is present in blastoids, the coronoid *Stephanocrinus* (Figures 2I, 2L, 5D), the flexible crinoid *Onychocrinus* (Tables 1, 3), and rarely in camerate crinoids such as in *Cyttarocrinus* (Figure 3Q, R). The pattern of cover plates on the tegmen of most monobathrid camerates, such as *Marsupiocrinus* (Figure 3O, P) is consistent with PBS A4, although the oral plates are absent, presumably non-calcified as they were roofed over by fixed cover plates within a mosaic of interradial tegmen plates over the subtegmenal mouth.

**Table 3:** Key oral region characters; ordered by Peristomial Border System (PBS).

Genus	PBS	PPCP*	Floor Plates	Periproct in CD interarea of oral region
**EDRIOASTEROIDEA**				
*Kailidiscus*	A1	moveable	quadriserial	Yes
*Cambraster*	A2	moveable	biserial	Yes
*Edriophus*	B1	moveable	biserial	Yes
*Astrocystites*	B1?	fixed	biserial	Yes
*Anedriophus*	B2	moveable	uniserial	Yes
*Isorophusella*	B2	moveable	uniserial	Yes
*Hypsiclavus*	B2	moveable	uniserial	Yes
**BLASTOZOA**				
*Ascocystites*	A2	moveable	biserial	No, lower on calyx
*Lepadocystis*	A2	moveable	biserial	No, in BC on calyx
*Quadrocystis*	A2	moveable	biserial	No, in BC on calyx
*Protocrinites*	A2	moveable	biserial	Yes
*Rhopalocystis*	A3	moveable	biserial	Yes
*Eumorphocystis*	A3	moveable	biserial	Yes
*Columbocystis*	A3	moveable	unknown	No, in BC on calyx
*Stephanocrinus*	A4	fixed	absent?	Yes
*Lepidocystis*	B2	unknown	uniserial	Yes
*Sinoeocrinus*	B2	unknown	Absent	High on calyx, defines CD interarea
**CRINOIDEA**				
*Hybocrinus*	A3	moveable	absent, non-calcified?	Yes
*Carabocrinus*	A3	moveable	absent, non-calcified?	Yes
*Palaeocrinus*	A3	moveable	absent, non-calcified?	Yes
*Illemocrinus*	A3	moveable	absent, non-calcified?	Yes
*Nuxocrinus*	A3	moveable	absent, non-calcified?	Yes
*Onychocrinus*	A4	moveable	biserial	Yes
*Cyttarocrinus*	A4	moveable	absent, non-calcified?	Yes
*Marsupiocrinus*	A4?	fixed	absent, non-calcified?	Yes
*Collicrinus*	A4?	fixed	absent, non-calcified?	Yes
*Elegantocrinus*	A4?	fixed	absent, non-calcified?	Yes
*Neoplatycrinus*	A4?	fixed	absent, non-calcified?	Yes

* Primary Peristomial Cover Plates.

**Figure 6:**
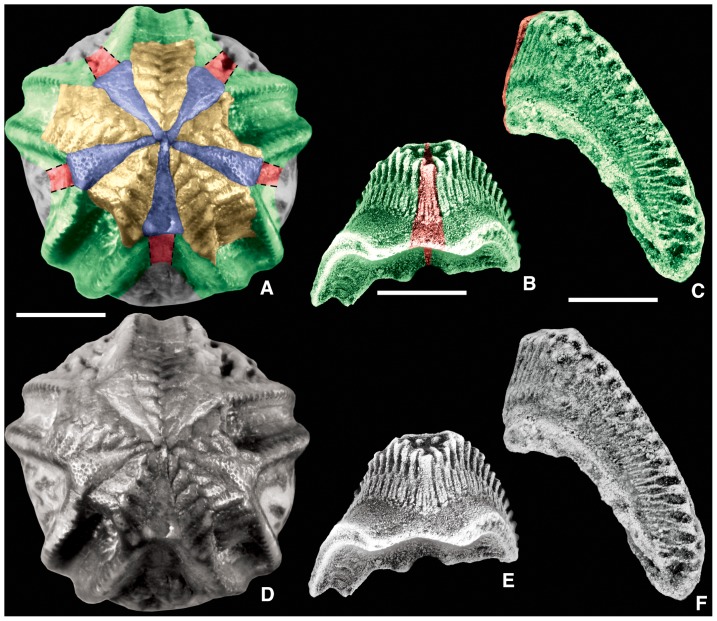
Homologous oral-region plates in the Middle Ordovician edrioblastoid *Astrocystites*. Colored and uncolored views for plate comparison; color key as in Figure 1. A, D. the edrioblastoid *Astrocystites ottawaensis* Whiteaves, 1897 [91], GSC 752, note the small orals encompassed within the larger “deltoid” plates formed by fused floor plates, and the suturing of the proximal ACP to the PPCP; B, C, E, F. isolated “deltoid” plates of *Astrocystites* sp. consisting of a small oral and floor plates fused together, B, E, oral view with peristome down, SUI 134871; C, F, lateral view with peristome upper left, SUI 134872. Scale bars: 5 mm (A, D), 2.5 mm (B, C, E, F).

In PBS B1, the peristomial border is formed from radially-positioned oral frame plates that are bordered by oral plates (Figure 5E). The associated floor plates are biserial. This type occurs with certainty only in Edrioasteridae such as *Edriophus* (Figures 4G, H, I, J, 5E). It likely also is present in edrioblastoids, such as *Astrocystites* (Figure 6A, D), rhenopyrgids and cyathocystids based on phylogenetic arguments; but the internal oral frame plates have not been directly observed. Small facets along the mouth edge of isolated *Astrocystites* orals hints at the presence of oral frame plates, but these have not been directly observed (Figure 6E). However, these oral plates in *Kailidiscus* and *Edriophus* are different by being smaller and, thus, not bearing the food groove into the peristome, which was done by the radially positioned oral frame plates. In *Cambraster* the food groove is along the oral plate sutures [47].

In PBS B2 the peristomial border is formed exclusively from radially-positioned oral frame plates presumably derived from modified floor plates (Figure 5F). This type is present in plesiomorphic eocrinoid taxa such as *Lepidocystis* ([9], pls. 1–3; Figure 4M, N), the eocrinoid gogiid *Sinoeocrinus* (Figure 4 C, D), *Gogia* [49], and many of the edrioasteroid clades, especially the isorophids [38], [50], [51]. Here five radial elements form around a bean-shaped peristomial opening, and an extra plate fits in the CD interray that does not bear an ambulacrum. In most cases floor plates extend from the oral frame plates forming the distal food grooves; but in at least one gogiid, *Sinoeocrinus*, brachioles are sutured directly to the oral frame plates ([52], fig. 4.1; Figure 4C, D). The edrioasteroid condition is also different in that the hydropore/gonopore structures are incorporated into the oral frame [50], [53], whereas they are located in the proximal interambulacral fields in plesiomorphic eocrinoids [9].

In derived blastozoans and plesiomorphic crinoids (those with an oral surface) the mouth frame is directly sutured to the calyx, producing an overall inflexible theca. There are typically few or no interambulacral plates on the oral surface. In plesiomorphic blastozoans, such as *Lepidocystis*, and plesiomorphic edrioasteroids the mouth frame is centered on a relatively flexible surface with numerous interambulacral plates. In more-derived edrioasteroids, such as edrioasterids and the edrioblastoid *Astrocystites* (Figure 6A, D), the oral frame is part of a rigid theca because the interambulacral plating is more strongly sutured.

#### Peristome and cover plates

The mouth (or peristome) in many blastozoans, plesiomorphic crinoids, and edrioasteroids is covered by presumably moveable cover plates, based on their size and arrangement, and the fact that they are commonly not preserved in place. The primary peristomial cover plates (PPCP) are interradially positioned and mark the branching points of the ambulacral perradial suture of the cover plate system (Figure 1). In many taxa there is a set of cover plates positioned along the shared ambulacra over the peristome, between PPCP 1, 3, 4 and the lateral PPCP 2, and 5, termed shared cover plates. When present these may either be differentiated or undifferentiated from the PPCP and the more distal ambulacral cover plates (ACP). The PPCP are the first cover plates formed during ontogeny in edrioasteroids [40], where they previously have been termed orals and lateral bifurcation plates. Later, shared cover plates are inserted between the lateral PPCP (plates 2 and 5) and the centrally positioned PPCP (plate 1, 3, and 4) as in *Isorophusella* [40]. This same pattern also occurs in *Lepadocystis* (Figure 1), *Rhopalocystis* (Figure 2A, B, D, E), *Eumorphocystis* (Figure 2G, H), and the cladid crinoids *Carabocrinus* (Figure 3A, G) and *Nuxocrinus* (Figure 3B, C). In some taxa the PPCP are undifferentiated from other cover plates and can be identified only by position, as in *Hybocrinus* (Figure 3H, I). However, greatly enlarged and clearly differentiated PPCP occur in the edrioblastoid *Astrocystites* (Figure 6A, D), the coronoid *Stephanocrinus* (Figure 2I, L), and many camerate crinoids, such as *Marsupiocrinus* (Figure 3O, P), *Collicrinus* (Figure 3D, J), *Elegantocrinus* (Figure 3E, F) and *Neoplatycrinus* (Figure 3M, N). These enlarged PPCP are commonly fixed as in coronoids and camerates. Two special cases are noted. In *Astrocystites*, proximal ambulacral cover plates suture to the PPCP rather than to either the oral plates or the floor plates (Figure 6A). The cladid crinoid *Illemocrinus* (Figure 3K, L) has 10 paired, moveable PPCP, with two cover plates centered on each of the five orals.

#### Floor plates

Ambulacral floor plates occur in all edrioasteroids and most blastozoans.

In edrioasteroids there are two non-homologous sets, a biserial adradial (inner) set and a biserial abradial (outer) set [48], [53]. The abradial set is in line with the oral plates of *Kailidiscus* (Figure 4A, B, E, F) and has a broad expression abradially from the cover plate articulation, suggesting that the oral plates are of abradial floor plate origin. These are the floor plates present in edrioasterids and *Cambraster* [47]. The adradial set forms the base of the food groove and the peristomial border suggesting that the oral frame plates and uniserial floor plates of isorophids are of adradial plate origin [53]. In derived blastozoans, floor plates are biserial and have a wide expression abradial to the cover plate articulation suggesting they are of abradial plate origin.

Floor plates are rare, but not unknown in crinoids. Biserial floor plates are present along the arms of some of the oldest known crinoids, including the possible cladid *Apektocrinus ubaghsi* [16], and the camerate *Glenocrinus globularis* [11], [16]. They are also present on the oral surfaces of at least two taxa of flexible crinoids, the Silurian-age *Homalocrinus liljevalli* ([54], pl. 6, fig. 15b) and the Mississippian-age *Onychocrinus ulrichi* ([54] pl. 67, fig. 8), which are the only non-Ordovician crinoids known to have calcified floor plates. The floor plates apparently supported the ambulacra across the flexible oral surface and onto the free arms. The highly regular biserial floor plates in both these taxa are aligned with the oral plates and do not appear to have been newly evolved. Rather they may be an atavistic character, suggesting that at least some crinoid lineages retained poorly calcified or non-calcified floor plates that were then calcified in these flexibles.

In most blastozoans, the floor plate series begins at the distal margins of the orals typically forming a biseries beginning on the left side of each ambulacrum. These floor plates bear the food groove and attachment structures for brachioles that may attach in a variety of different styles to the floor plate series [12]. In most taxa these plates are part of the calyx wall, as in *Rhopalocystis*; whereas in others the floor plates may extend epithecally outward and downward across the calyx, as in many glyptocystitoids. In blastozoans with erect ambulacra (not simply brachioles arising from floor plates), such as hemicosmitoid *Caryocrinites* [55] and the diploporan *Eumorphocystis* [56], the appendages are biserial and continuous with the recumbent floor plates, suggesting they are elevated floor plates, an extension of the axial skeleton [57].

#### Coelomic canals and erect ambulacra

A key synapomorphy for crinoids is possession of arms with coelomic canals. Arm (or brachial) plates are extensions of extraxial skeleton of the calyx and in living crinoids include coelomic canals. Most fossil crinoids are presumed to have had coelomic arm extensions as suggested by the large thecal arm openings and commonly deep brachial grooves to contain the coelomic extensions [20]. Coelomic canals also appear to be present in the diploporan *Eumorphocystis* (Sumrall, unpublished data), and possibly other blastozoans still under study.

### Extraxial Skeleton in the Oral Region

The following morphologic features are common on the oral surface, yet are part of the perforate extraxial skeleton as defined by Mooi and David [1], [2]. The majority of the perforate extraxial skeleton is comprised of thecal plates, which are beyond the scope of this study, as is the imperforate extraxial skeleton comprising the column or stalk of pelmatozoans.

#### Hydropore and gonopore

The hydropore and gonopore structures of plesiomorphic echinoderms lie in the proximal right interambulacral fields unrelated to the plating of the peristomial border. This occurs in the eocrinoids *Kinzercystis* ([9], pl. 6 fig. 3) and *Gogia* (Sumrall pers. obs., 1995) and the edrioasteroid *Kailidiscus* [48]. In these taxa the hydropore and gonopore structures are closely positioned and in the form of small pyramids or, in the case of one of the structures in *Kinzercystis*, a small polyplated spout.

In more-derived taxa these structures migrate phylogenetically into the peristomial border. In some edrioasteroid-grade taxa and derived blastozoans, the hydropore and gonopore structures are positioned within the oral plates of the CD area (Figures 1, 4G). Commonly there are three orals, but reductions to two or one occur among various taxa [3], [4]. The hydropore and gonopore structures are typically in the form of a slit crossing a plate suture (commonly interpreted as a hydropore) and a pore covered with a small pyramid (commonly interpreted as a gonopore). In crinoids, the hydropore, if present, is in the single CD oral (Figure 3A, B, H). The gonopore structure has not been confidently observed in Paleozoic crinoids.

In isorophid edrioasteroids these structures are differently incorporated into the peristomial border, presumably because of the absence of oral plates. Here, modified oral frame plates along the C ambulacrum house two internal openings that lead to a single external vent [50], [51]. The external structure is formed from a series of between one and many small hydropore oral plates that may be modified from the cover plate series [38], [50], [58].

#### Periproct

The periproct is located in the CD interambulacral area, except in a few clades, such as glyptocystitoids, where placement in the BC interradius is clade diagnostic [39]. In edrioasteroid-grade taxa and most blastozoans, the periproct is uniformly in the form of a pyramid composed of a complex series of lathe-shaped plates around a somewhat circular opening. This structure sits in a field of interambulacral plates, but where these plates are reduced or absent, as in crinoids, the periproct may be in contact with the peristomial border (Figure 3B, H, K, Q), or part of the rigid tegmen (Figure 3D, O).

#### Interambulacral plates

Interambulacral plates occur in plesiomorphic pentaradiate echinoderms and form a plate series between the ambulacra on the oral surface. These plates are the so-called perforate extraxial skeleton of Mooi and David [1], [2]. In *Kailidiscus* these plates form a somewhat flexible cover on the upper side of the theca but lack pores (Figure 4A). In *Lepidocystis*, *Kinzercystis*, some gogiids, and *Cambraster* they are perforate and form the boundary of the oral surface. In *Lepidocystis*, *Kinzercystis* they are differentiated from plates lower in the theca by a transition to more imbricate plates. In most advanced blastozoans and hybocrinid and primitive cladid crinoids, interambulacral plates are lost or absent, whereas in other taxa, such as edrioasteroids they are the dominant plate type of the theca. The interambulacral plates on the tegmens of many camerate crinoids are presumably independent in origin as they are required to fill the space between the fixed ambulacral cover plates (Figure 3O).

## Discussion

This study provides new criteria for recognizing homologies between blastozoans, crinoids, and edrioasteroids. The similar suites of oral region characters present in these groups has previously gone unrecognized, probably because oral surfaces in crinoids have generally not been studied, and edrioasteroids had their own morphologic terminology [38]. Preserved oral surfaces in crinoids have been treated as curiosities, mostly because they are uncommon [25], and are typically not incorporated in crinoid systematics, where the focus has been almost exclusively on calyx plate patterns [59].

Definition of oral characters and possible homologues is best done by comparison with the two model blastozoan taxa: the glyptocystitoid rhombiferan *Lepadocystis* (Figure 1) and the eocrinoid *Rhopalocystis* (Figure 2A, B, D, E).

In the arrangement of the oral plates, *Lepadocystis*, like other glyptocystitoids, has Peristomial Border System A2, which also occurs in the edrioasteroid *Cambraster* (Table 3). Peristomial Border System A3, as in *Rhopalocystis*, is also present in *Eumorphocystis* (Figure 2G, H) the paracrinoid *Columbocystis* (Figure 2J, K) and plesiomorphic crinoids such as *Hybocrinus* (Figure 3H, I), *Carabocrinus* (Figure 3A, G) and *Nuxocrinus* (Figure 3B, C); but it is absent in edrioasteroids. Peristomial Border System A4 is a pseudo-pentaradiate arrangement, with just the five PPCP covering the peristome, which appears to have evolved independently in coronoids and some crinoids. The coronoid *Stephanocrinus* (Figure 2I, L), the flexible crinoid *Onychocrinus*, and many camerate crinoids, such as *Collicrinus* (Figure 3D, J), *Neoplatycrinus* (Figure 3M, N), and *Marsupiocrinus* (Figure 3O, P) all have fixed PPCP over the peristome. However, the orals are part of the high coronal processes in coronates, they were present on the flexible integument covering the orals surface of flexible crinoids ([54], pl. 67, figs. 7, 8), whereas they are absent from the tegmen of camerates and were apparently non-calcified and associated with the subtegmenal mouth. Thus, it is clear that Peristomial Border System A4 is homoplasic, or convergent, in these groups (Table 3). Both *Rhopalocystis* and *Lepadocystis* have three orals (O1, O6, O7) in the CD interray, which also occurs in many diploporans and the edrioasteroids *Cambraster* (as IIP) and probably *Kailidiscus*. The three orals are reduced to only one in crinoids, the eocrinoid *Ascocystites* ([9], text-fig. 30), and the diploporan *Eumorphocystis* (Figure 2G), through either loss or fusion. True orals are absent in isorophids (Figure 4K, L), *Lepidocystis* (Figure 4M, N), *Sinoeocrinus* (Figure 4C, D), and *Gogia* [49].

Both *Lepadocystis* and *Rhopalocystis* have clearly defined PPCP and additional intercalated SCP that are only slightly larger than the ACP, a pattern also in edrioasteroids (the orals and lateral bifurcation plates of Bell [38]) and plesiomorphic crinoids. A more derived condition is to have the PPCP greatly enlarged as in Peristomial Border System A4. Because the PPCP form before other peristomial cover plates, the presence of only five large PPCP suggests paedomorphosis, as in *Neoplatycrinus* (Figure 3M, N).

Both *Lepadocystis* and *Rhopalocystis* have a peristomial border directly sutured to a rigid thecal or calyx wall, which is typical for all blastozoans and crinoids. There are no interambulacral plates between the floor plates that underlie the ambulacral rays. This makes for a more rigid theca overall than the theca of many edrioasteroids such as *Kailidiscus*, *Cambraster*, and *Isorophusella*, all of which had the mouth frame in a loosely sutured, or placed within an imbricated, field of interambulacral plates between the floor plates of adjacent ambulacra. Evolution of a more rigid theca occurred more than once as indicated by edrioasterid and edrioblastid thecae, neither of which is thought to be the origin of the rigid blastozoan thecae.

Floor plates provide a substrate for the ambulacra in edrioasteroids and blastozoans. In *Lepadocystis* and *Rhopalocystis* the floor plates had facets for insertion of small biserial brachioles that passed food to the ambulacra. Some blastozoans, such as *Ascocystites* and *Eumorphocystis*, had erect ambulacra analogous to crinoid arms and unlike the small, thin brachioles of most blastozoans. In *Ascocystites* these larger “brachioles” ([9], text-fig. 30) are direct extensions of the ambulacral grooves that branch on the oral surface leading to numerous (20–30), erect, non-branching appendages constructed from biserial floor plates extending from the oral surface. In *Eumorphocystis*, the floor plates in the “arms” remain biserial but carry with them a plate series from the thecal wall with a coelom between. Uniserial brachioles arise from the floor plates alternately [56]. However, the floor plates on the oral surface give rise to standard biserial brachioles.

In crinoids, the erect ambulacra are elevated on branching arms composed of brachial plates, which are uniserial in early crinoids [16]. The uniserial brachial plates are radial in position and may have evolved by extension of the radial series of plates on the theca, such as those in the eocrinoid *Rhopalocystis* where a straight line of thecal plates extends from each of the five radials to the tips of each of the five ambulacra ([60], fig. 293). Thus, brachial plates apparently originated from the extraxial skeleton, whereas floor plates in blastozoans originated from the axial skeleton. Crinoid arms may be a combination of the axial (ambulacra) and extraxial systems (brachials) as in *Eumorphocystis*. Floor plates are rare in crinoids [16] and were presumably lost or non-calcified in most crinoids because they were redundant. In plesiomorphic crinoids the ambulacra run along the oral plate sutures before extending up the arms, thus floor plates are unnecessary for support of the ambulacra. However, the flexibles *Onychocrinus* ([54] pl. 68, fig. 8) and *Homalocrinus* ([54] pl. 6, fig. 15b) exhibit apparent atavistic restoration of floor plates in order to support the ambulacra across the flexible oral surface. Presumably floor plates were lost or non-calcified in other crinoids because the arm brachial plates supported soft tissues of the erect ambulacra and the underlying coelomic extensions [20]. This is not unprecedented within blastozoans as numerous examples in several clades exist of ambulacra not being supported by calcified floor plates. Examples include diploporans such as *Glyptosphaerites*, *Eucystis* and *Fungocystites* where food grooves extend across thecal plates unsupported by ambulacra [61]. Further, some blastoids bearing wide ambulacra, such as *Pentremites*, bear the food groove on the lancet plate of extraxial origin [4].

Presumably there was an evolutionary advantage to elevating the ambulacra in order to increase the size of the food-gathering apparatus, as it evolved multiple times [12], [62]. Several clades of blastozoans extended the floor plates of the axial skeleton above the theca, whereas crinoids and *Eumorphocystis* extended the thecal plates of the extraxial skeleton to form arms. Only crinoids consistently developed branching of these erect structures, which may be why they prevailed and blastozoans did not. [But see the branching “arms” of the gogiids *Lyracystis* [63] and *Balangicystis* [64].

Crinoids are united in having coelomic canals extending up their arms [16] which are lacking in the erect brachioles mounted on the ambulacral floor plates of most blastozoans [10]. Yet, in the diploporan *Eumorphocystis* there are arm-like appendages that appear to have coelomic openings to the theca (Sumrall, pers. obs.). The Middle Cambrian rhombiferan *Dibrachicystis* exhibits a large coelomic lumen in each of its two arms [65]. Although these particular blastozoans may not be closely related to crinoids, they do demonstrate the evolution of coelomic extensions into feeding structures in more than one clade of pelmatozoans. Thus, crinoid arms may have homologies shared by some blastozoans [66], although this viewpoint has been strongly debated [20]. Nevertheless, it shows that coelomated arm structures found in the arms of crinoids are not unique to the clade and that blastozoans were capable of evolving analogous structures.

### Homologies Shared by Blastozoans and Crinoids

Data presented herein (Table 3; Figures 1, 2, 3, 4, 5) unambiguously demonstrate shared homologies between blastozoans and crinoids. Those crinoids that share the greatest number of homologous character states with blastozoans are the plesiomorphic hybocrinids and early cladids (cyathocrinids). The camerate crinoids share some key homologies but also have evidence for newly derived characters, notably the rigid tegmen over the mouth. Flexibles are derived from later cladids [34] and will not be considered here. Disparids have a reduced suite of oral characters and will be treated in a later paper.

Plesiomorphic crinoids, like most blastozoans, construct a peristomial border from oral plates. With only one oral in the CD interray, they have five orals total. Many blastozoans have two or three orals in the CD interray, as does the edrioasteroid *Cambraster* [47]. The apparent reduction to only one CD oral is common in oral-plate-bearing echinoderms as in the eocrinoid *Ascocystites* and the diploporan *Eumorphocystis*.

The plesiomorphic crinoids *Hybocrinus*, *Carabocrinus*, *Illemocrinus*, and *Nuxocrinus* (Figure 3) have PBS A3 (Table 3), also present in the eocrinoid *Rhopalocystis*, the diploporan *Eumorphocystis*, and the paracrinoid *Columbocystis*. The more derived camerate crinoids are questionably placed in PBS A4 (Table 3) because the five PPCP are fixed in the center of the tegmen, such as in *Marsupiocrinus*, *Collicrinus*, and *Neoplatycrinus* (Figure 3). Most camerates have no evidence of the orals because they have a tegmen composed of fixed plates that cover the subtegmenal mouth. However, rarely the orals are preserved, as in the monobathrid *Cyttarocrinus* (Figure 3Q, R), supporting the hypothesis that camerates evolved from an ancestor with orals. *Cyttarocrinus* apparently had moveable PPCP, as in typical plesiomorphic crinoids.

Moveable primary peristomial cover plates are in most of the blastozoans discussed herein, and in the plesiomorphic crinoids and *Cyttarocrinus* (Table 3). Each of the five PPCP is interradial and project from the central interradial edge of an oral. They can be identified during early ontogeny in edrioasteroids and blastozoans [3], and they are homologous with the fixed PPCP in camerates. The cladid *Illemocrinus* (Figure 3K, L) is unique in having two PPCP projecting from the edge of each oral, for a total of 10 PPCP, a feature not present outside the cladids.

Plesiomorphic crinoids have a hydropore opening through the CD oral, similar to derived blastozoans. A gonopore opening, sometimes present in blastozoans and edrioasteroids, has not been recognized in any crinoids. The periproct is also in the crinoid CD interray as it is for all edrioasteroids, the eocrinoid *Rhopalocystis*, the diploporan *Eumorphocystis*, and the coronate *Stephanocrinus*. Glyptocystitoid and paracrinoid blastozoans have the periproct on the BC interray lower on the theca (Table 3).

### Homologies Shared by Blastozoans and Crinoids with Edrioasteroids

Some of the oral characters in crinoids and blastozoans have homologues in edrioasteroids, but more importantly, edrioasteroids have the widest range of oral region morphologies, including four types of peristomial border systems and three types of floor plates (Table 3). *Kailidiscus* has quadriserial ( = double biserial) floor plates [48] (Figure 4A, B, E, F). The abradial (outer) row of floor plates in *Kailidiscus* is probably homologous with the biserial floor plates of *Cambraster* and edrioasterids [47], [53]. *Kailidiscus*, *Cambraster*, and other Cambrian edrioasteroids such as *Totiglobus* and *Aragocystites* [47], [48], [67], [68], have plates that have been termed the integrated interradial plates, or interradial mouth plates, that are oral plates homologous with those in crinoids and blastozoans. Whereas these oral plates form the mouth frame of Peristomial Border System A in these edrioasteroids, blastozoans and crinoids, *Kailidiscus* is an exception because it has an inner row of plates between the peristome and the orals that give rise distally to the adradial set of biserial flooring plates (Figure 4A, B, E, F).

Isorophids have a very different pattern, with Peristomial Border System B, in which oral frame plates (radial elements) construct the mouth frame (Figure 4K, L). These uniserial elements also build the uniserial floor of the ambulacra distally. The radial uniserial oral frame plates have equivalents in edrioasterids, but the latter have biserial, rather than uniserial, distal floor plates. Oral frame plates are totally absent in other Cambrian edrioasteroids such as *Aragocystites*, *Stromatocystites* and *Totiglobus*. It is unclear if these uniserial elements derive from the fusion of the inner row of floor plates that border the oral frame in *Kailidiscus*. A noteworthy observation is that this construction of the mouth frame incorporating oral frame plates is also present in at least three Cambrian blastozoans, *Lepidocystis*, *Sinoeocrinus*, and *Gogia* [9], [49], [52].

### Is it all Plesiomorphy?

It has been argued that the identification of these homologous elements simply represents deep-seated plesiomorphy of the clade of pentaradiate Echinodermata [69]. This is a Straw Man argument that overstates the nature of plesiomorphy. All synapomorphy is based in homologous changes in plesiomorphic conditions. That UEH identifies deep-seated homology means that these features are plesiomorphic in origin. But it is the transformations of these plesiomorphic characters into derived states that provide characters for phylogenetic inference and phylogenetic tests for homologous transformation.

More importantly, the identification of six different types of mouth frame construction, or Peristomial Border Systems (PBS), defined among edrioasteroids, blastozoans, and crinoids (Table 3) demonstrates that there is no single symplesiomorphy of mouth frame construction shared by all groups. Type A bearing oral plates and type B bearing oral frame plates are minimally two separate non-homologous constructions of the border of the peristome. Edrioasteroid-grade taxa have the widest range of mouth frame types indicating separate derivation of at least the oral frame plates (type B). Regardless, there are no edrioasteroids that have plesiomorphic mouth frame reconstructions that can be homologized to those present in crinoids. They are either too derived as in edrioblastoids and edrioasterids, or are non-homologous as in isorophids.

Mouth frames constructed from both interradial oral plates and radial biserial floor plates (from the adradials) (PBS A1) are occur only in *Kailidiscus*. Mouth frames constructed by laterally separated orals (PBS A2) are in *Cambraster* and similar edrioasteroids, certain eocrinoids (e.g., *Ascocystites* [70]), and glyptocystitoid blastozoans. Mouth frames where all orals are in lateral contact around the peristome (PBS A3) occur only in certain eocrinoid (e.g., *Rhopalocystis*) and diploporan (e.g., *Eumorphocystis*) blastozoans, and in plesiomorphic crinoids. Mouth frames formed exclusively from uniserial floor plates (PBS B2) are only in isorophid edrioasteroids and certain imbricate (e.g., *Lepidocystis*) and gogiid (e.g., *Sinoeocrinus*) blastozoans. The edrioasterid edrioasteroids incorporate elements of both PBS A2 and PBS B2 in their oral frame construction and are referred to PBS B1. PBS A4, where the five primary peristomial cover plates (PPCP) all meet in the center, is judged to be an evolutionary grade, which occurs in coronates and camerate crinoids.

Edrioblastoids (e.g., *Astrocystites*, Figure 6A) have only the tips of the 5 PPCP meeting in the center, which is very different from the shared lateral PPCP sutures in coronates and camerates. Other differences can be noted. Although edrioblastoids such as *Astrocystites* do have oral plates, the nature of these plates is entirely different to the condition present in crinoids and other pentaradiate taxa. *Kailidiscus* and the edrioasterid clade (edrioasterids, edrioblastoids, cyathocystids and rhenopyrgids) have podial pores in the oral plates. Podial pores wrap around the oral plates suggesting that in these taxa oral frame plates are present although this has only been documented in edrioasterids [38]. On this basis, *Astrocystites* is questionably placed in PBS B1 with the edrioasterids (Table 3). In crinoids and derived blastozoans these pores are not present, and there is no indication of oral frame plates suggesting a fundamentally different configuration of the peristomial border.

Furthermore, the autapomorphies in edrioblastoids make them unlikely to be the stem group from which crinoids evolved. Edrioblastoids lost the plesiomorphic 2-1-2 ambulacral symmetry with shared ambulacra, instead having pseudo five-fold symmetry (sensu Sumrall and Wray [39]). In contrast, plesiomorphic crinoids have well developed 2-1-2 symmetry with shared ambulacra. Edrioblastoids have floor plates fused to the oral plates forming large “deltoids” (Figure 6A, B, C) whereas crinoids do not.

Although the phylogenetic meanings of the plating in the various peristomial border types has not yet been fully documented, a few observations suggest a path forward. It can be easily argued that edrioasteroids are a paraphyletic grade of organization that may have led to several branches in later echinoderm evolution [48]. However much of this depends on the polarity of oral plates and oral frame plates in a phylogenetic sense.

If something akin to *Kailidiscus* had the plesiomorphic state for pentaradiate echinoderms, then oral plates are plesiomorphic and later lost in Imbricata, Gogiida, and Isorophida where a new type of oral frame plates likely formed by fusion of floor plates into radial elements. Edrioasterids, with both orals and oral frame plates, are inferred to be the outgroup to the clade containing edrioblastoids, cyathocystids, and rhenopyrgids [22], [23], [71]. For crinoids to be derived from edrioblastoids, the oral frame plates must be lost in this scenario.

If something akin to the imbricate eocrinoid *Lepidocystis* has the plesiomorphic condition for pentaradiate echinoderms, then the oral frame plates of Imbricata, Gogiida, Edrioasterida and Isorophida are plesiomorphic. This would require the non-homology of oral plates in crinoids and derived blastozoans, if crinoids were separately derived from edrioblastoids, which seems unlikely based on evidence presented herein. Furthermore, crinoids derived from edrioblastoids would require a secondary loss of oral frame plates and numerous homoplasic modifications of the nature of the oral plate system, which would generate a system that is much less parsimonious than if crinoids were derived from within derived blastozoans.

Thus, we reject the hypothesis [69] that oral region characters are all similar in early pentaradial echinoderms, reflecting a deep plesiomorphy. Rather, there is a wide range of oral region homologous characters available for future phylogenetic analyses.

## Conclusions

Previous studies have hypothesized independent origins of crinoids and blastozoans within the Echinodermata. Any similarities between these groups were attributed to either homoplasy or plesiomorphy shared by a wide range of primitive echinoderms. Applying the Universal Elemental Homology (UEH) approach of Sumrall [3] and Sumrall and Waters [4], we present numerous examples of homologous characters in the oral regions of crinoids and blastozoans, as well as edrioasteroids. The oral regions of these taxa all contain a relatively complex suite of functionally-integrated morphologic characters, with a wide variety of different plate types and character states that compose the peristomial and ambulacral systems (Tables 2 and 3). Such similarities in complex systems are unlikely to have arisen independently.

The peristome (or mouth) is constructed by plates that form the Peristomial Border System (PBS). Two major groups, with six different total types of peristomial border systems are recognized.

PBS type A is constructed exclusively from interradially-positioned oral plates and occurs only in plesiomorphic edrioasteroids and certain blastozoans, as well as crinoids (Table 3). PBS A1 and A2 are only in certain edrioasteroids and blastozoans, whereas PBS A3 and A4 are exclusively in crinoids and other blastozoans. There is no overlap in PBS types between crinoids and edrioasteroids.

PBS type B is constructed from radially-positioned uniserial oral frame plates, and may also include interradially-positioned orals. PBS type B occurs only in certain other edrioasteroids and blastozoans, and is absent in crinoids (Table 3). PBS B1 has both orals and oral frame plates and is only in edrioasterid edrioasteroids, which likely includes the edrioblastoids. PBS B2 has only oral frame plates and occurs in isorophid edrioasteroids and imbricate and gogiid eocrinoid blastozoans.

Recognition of separate axial and extraxial skeletal elements indicates that erect feeding structures (brachioles and “arms”) in blastozoans are mostly formed from the axial skeleton floor plates, whereas in crinoids erect feeding structures (arms) are mostly composed of extraxial thecal plates.

By clearly documenting and defining the homologous characters of the oral region, including the various types of peristomial border systems, we provide a fundamental set of characters for future parsimony-based phylogenetic analyses, including both axial and extraxial skeletal elements, to explore the relationships between crinoids, blastozoans, and edrioasteroid-grade echinoderms. Such an approach is critical to producing well-supported hypotheses for the branching order among stem-group echinoderms necessary to assemble the echinoderm tree of life.
